# Behavioral health and generative AI: a perspective on future of therapies and patient care

**DOI:** 10.1038/s44184-024-00067-w

**Published:** 2024-06-07

**Authors:** Emre Sezgin, Ian McKay

**Affiliations:** 1 The Abigail Wexner Research Institute at Nationwide Children’s Hospital, Columbus, OH USA; 2 The Ohio State University College of Medicine, Columbus, OH USA; 3 Department of Psychiatry and Behavioral Health, Nationwide Children’s Hospital, Columbus, OH USA

**Keywords:** Psychology, Paediatrics, Information technology

## Abstract

There have been considerable advancements in artificial intelligence (AI), specifically with generative AI (GAI) models. GAI is a class of algorithms designed to create new data, such as text, images, and audio, that resembles the data on which they have been trained. These models have been recently investigated in medicine, yet the opportunity and utility of GAI in behavioral health are relatively underexplored. In this commentary, we explore the potential uses of GAI in the field of behavioral health, specifically focusing on image generation. We propose the application of GAI for creating personalized and contextually relevant therapeutic interventions and emphasize the need to integrate human feedback into the AI-assisted therapeutics and decision-making process. We report the use of GAI with a case study of behavioral therapy on emotional recognition and management with a three-step process. We illustrate image generation-specific GAI to recognize, express, and manage emotions, featuring personalized content and interactive experiences. Furthermore, we highlighted limitations, challenges, and considerations, including the elements of human emotions, the need for human-AI collaboration, transparency and accountability, potential bias, security, privacy and ethical issues, and operational considerations. Our commentary serves as a guide for practitioners and developers to envision the future of behavioral therapies and consider the benefits and limitations of GAI in improving behavioral health practices and patient outcomes.

## Introduction

Rapid advancements in the fields of artificial intelligence (AI) have reshaped the healthcare and clinical practice landscape, delivering solutions that optimize processes and have the potential to enhance patient outcomes^1^. These technological progress have led to a number of applications that address an extensive range of challenges encountered in healthcare. Such applications have introduced improvements in diagnostic accuracy and treatment planning^2–4^, have personalized medical interventions^5–7^, expedited drug discovery and development^8^, and broadened the reach of medical services through remote monitoring and telemedicine^9,10^.

As a subset of AI, generative AI (GAI) models have gained increasing interest from the public and research communities owing to their demonstrated proficiency and computational efficiency in task execution. GAI models are principally neural networks or machine learning algorithms specialized to identify the patterns and structures within a dataset in order to generate variations or new data or content. Diffusion models^11^, Generative adversarial networks^12^, and Transformer networks^13^ are some of the widely adopted GAI models used in industry and research. These, in particular, have emerged as powerful tools in clinical research and the healthcare industry, where they serve pivotal roles in domains such as medical data processing^14^, genomic simulations and therapeutics^15^, synthetic data generation^16^, and image generation for diagnostics^17,18^. The latter includes tasks such as high-resolution MRI reconstruction, image synthesis, contrast adjustment, and image enhancement, thereby broadening the scope of medical diagnostics^19^. Emerging at the forefront are medical foundation models, or multimodal foundation models, which combine various data types, including images, text, voice, and sensor data, to streamline clinical decision-making processes^20,21^. Their versatility and broad applicability indicate that these models could potentially redefine healthcare practices, communication, and documentation. However, with the recent developments in image generation models including diffusion models (recent examples: Stable Diffusion XL, Midjourney v5), generative adversarial networks, variational autoencoders^22^, and pre-trained transformers (e.g., DALL-E 3), there are new venues in healthcare to explore and leverage generated images in healthcare delivery.

## Gap in behavioral health practice

Despite these advancements, the application of generative models in the behavioral health domain remains underexplored. Recent studies illustrate the application of large language models (LLMs) in augmenting empathy in online peer support platforms^23^, supporting behavioral health services^24^, building GAI integrated chatbot to support patients^25,26^, and addressing behavioral health information seeking activities^27^. Of the many proposed uses of GAI in behavioral health^28^, image generation has yet to be explored^29^. Both including and expanding these capabilities, the utilization of a multimodal approach invites broader opportunities, especially with the inclusion of diverse data streams from personal health records, digital ecosystems, and medical records^20^.

Meta-analyses examining psychotherapeutic interventions have found equally strong effect sizes for many common treatment modalities (e.g., CBT for Depression; 0.7 effect size)^30^. However, when compared to control groups across several meta-analytic studies, higher variance has been found in intervention groups, suggesting heterogeneity in individual-level treatment effects. Aptitude by treatment interactions (ATIs)^31^, where an individual’s unique circumstances, needs, or characteristics are prioritized for optimal outcomes, may explain this variability^30,32^. Therefore, it is imperative to explore how AI models can be harnessed to augment treatment efficacy in evidence-based practices through the use of more personalized treatment components^30^. In particular, generative models could support the creation of idiosyncratic and contextually relevant exercises for patients, thereby making therapeutic interventions more targeted and potentially more effective. GAI shows increased promise in conjunction with human-in-the-loop and human-AI collaboration approaches^33,34^, which integrate human intelligence and feedback into the AI decision-making process^35^. In line with that, GAI could be effective across a variety of therapeutic interventions to better meet the individual’s unique needs and attributes^28,36^. Within this scope, we provide a new perspective on adopting GAI in behavioral health.

## Behavioral health and AI-based image generation

In clinical practice, images and visuals are often used to enrich conversation, aid in the learning process, and enhance therapeutic interventions^37,38^. Provider (e.g., psychiatrist, psychologist, counselor, therapist, nurse, and social worker) initiated verbal conversations with visual cues in healthcare settings have already been used in a number of strategic ways, often to improve treatment engagement^39^. Some examples include reducing emotional discomfort, improving patient education and learning, reducing healthcare disparities and stigma, increasing positive interactions between patients and providers, increasing personalization of treatment, and access to care^40^.

In light of the growing evidence supporting the potential of generative models for images in various healthcare applications and integration (e.g., radiology and synthetic data^19^), it is necessary to investigate their utility in behavioral health services. The integration of generative models for images into existing evidence-based therapies, such as cognitive-behavioral therapy (CBT)^41^, dialectical behavioral therapy (DBT)^42^, and acceptance and commitment therapy (ACT)^43^, could enhance the personalization and potential effectiveness of these interventions, ultimately leading to improved patient outcomes and patient-provider experiences. For example, an essential component of CBT includes teaching patients to modify their patterns of thinking (T), feeling (F), and behaving (B). In working with children who may struggle to understand the dynamic interaction between their thoughts, feelings, and behaviors, it may be useful to extend these interventions to include generated personalized images. These images may provide clarity and simplicity to a somewhat abstract concept, promote insight into one’s maladaptive patterns, and provide strategies for change. For example, using pictures to depict one’s tendency to catastrophize in social situations (T), often leads to worsening mood (F), and subsequent social isolation (B). A new “triangle” (CBT triangle showing how thoughts, feelings, and behaviors are connected) can be generated depending on the individual’s goals or progress to illustrate ways new actions (e.g., approaching a social activity) may help reduce aversive emotions (e.g., sadness), and promote more balanced beliefs (e.g., “I met some new people, and they did not seem to hate me.”).

As the literature is growing, and researchers are yet to study the impacts of this technology on behavioral health outcomes, we aim to provide a glimpse at the future to guide and inform practitioners and developers on how to yield the potential benefits of these innovative tools in clinical practice and patient care. As multimodal foundation models in medicine are growing with text, image, and audio^44^, focusing on image generation and visual output (rather than multimodal engagement) would be a practical first step in introducing GAI in behavioral health therapies at clinics toward envisioning clinical utility and human-AI collaboration. In the following use case, we discuss the use of GAI to augment therapies with a focus on instilling emotional empowerment in the pediatric population.

## Use case: emotional empowerment through the use of GAI-based images

**Fig. 1:**
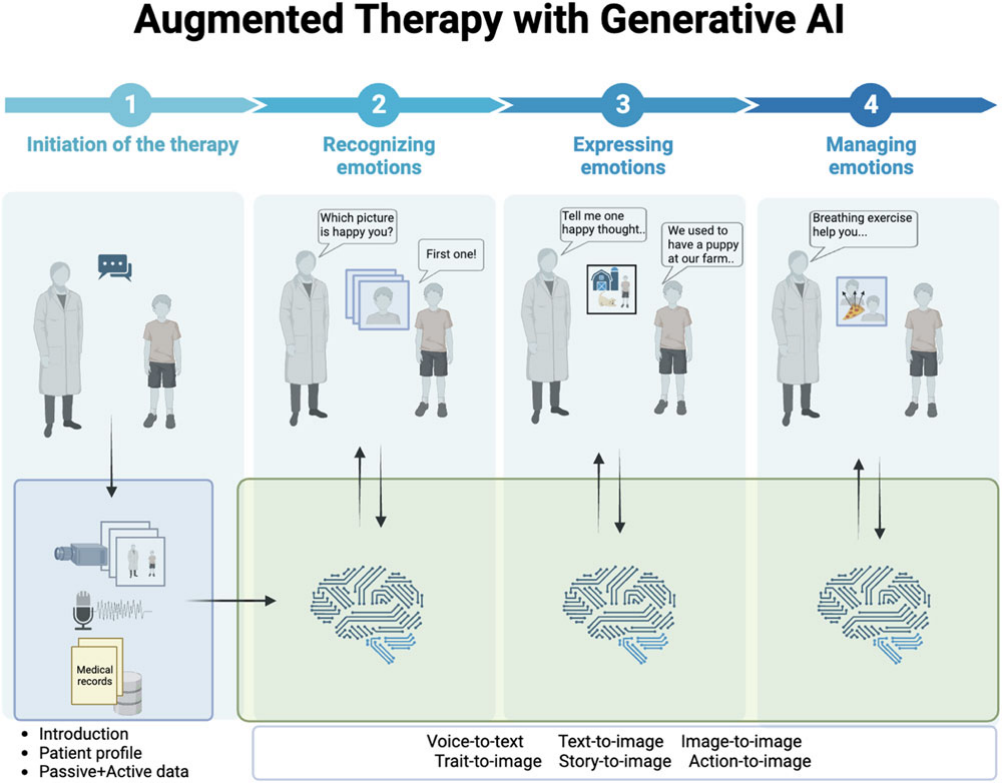
Augmented therapy with generative AI for image generation. Initiated with therapist and patient conversation, creating input including speech (prompts), visual (identifying setting, individuals, expressions), and medical records (clinician notes and patient history). Image, voice, and text inputs inform future prompts via conversations in a multi-agent relationship based on the prompt triggered (e.g., voice to text and then text to image by clinician’s prompt; AI is listening to the story and using patient visual traits to create relevant images to the story). In each session, the prompt could be redefined based on the user feedback and the conversation (e.g., to manage emotion, the clinician can define actions, and those could be displayed as images).

Emotional intelligence broadly refers to a person’s ability to navigate and comprehend the intricate world of emotions, both within oneself and in others. As a practical application of emotional intelligence, emotional empowerment involves the employment of emotional intelligence skills in one’s daily life. Research has demonstrated the importance of fostering emotional empowerment in personal development and levels of well-being^45^. Furthermore, research has demonstrated a relationship between the fostering of emotional empowerment in children and positive life outcomes, including enhanced resilience, school success, social skills, attention and concentration, decision-making abilities, and reduced long-term mental and behavioral health symptoms^46^. The ability to understand and control one’s emotions is crucial for children to navigate various challenges and complexities in life, and emotional empowerment can be a valuable tool for achieving this. We envision that the use of GAI models during pediatric behavioral therapies can augment the therapeutic process and contribute to promoting emotional empowerment. Figure 1 highlights “augmented” 3-step emotional empowerment therapy sessions with GAI.

### Step 1: Recognizing emotions

The first step of emotional empowerment consists of developing an ability to recognize and name emotional states. The fostering of these skills in early years is vital for ones’ interpersonal functioning over the course of their lifespan^47^, and has been inversely related to the development of psychological and behavioral challenges in both children and adults^48^. These skills are also a necessary prerequisite to experiencing positive outcomes from the delivery of many therapeutic interventions^49^.

**Fig. 2:**
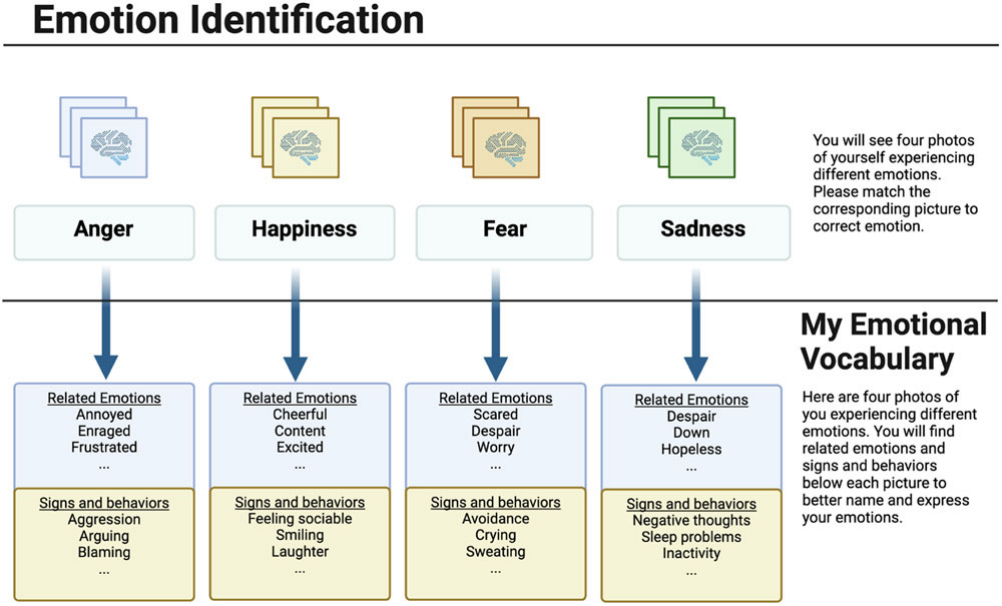
Emotion identification card. The card is including generated images of a patient reflecting anger, happiness, fear and sadness. Instructions are first asking patient to match images with emotions. Then, it guides to match images with corresponding emotional vocabulary items.

Giving children opportunities to recognize a wide range of emotions in themselves and in others as well as scaling intensity may help support the acquisition of these skills early in life because children often learn better through visual cues and illustrations^50^, GAI models may enhance learning through visual depictions of various facial expressions associated with different emotions. These images could serve as reference points for children to understand and recognize specific emotional states like happiness, sadness, anger, surprise, and more. This tool can be further personalized and more engaging via gamification, which has been broadly defined as the application of gamifying elements for treatment or intervention purposes^51,52^. Prior use of these strategies to target emotional identification deficits has been related to improved emotion recognition and feedback among the pediatric population^53^. To expand on this work, applications with image generation tools can present a series of personalized images (self or someone familiar) displaying different facial expressions, allowing children to match them with corresponding emotions (see Fig. 2). This interactive approach could make the learning process more enjoyable and engaging for children. Moreover, GAI models can be used to create personalized content that aligns with the child’s cultural background, age group, or specific needs. By generating images that resemble the child’s own environment, ethnicity, or familiar characters, the GAI can provide relatable visual cues that facilitate improved emotional understanding and identification. Please see https://github.com/edsz7/supplementary, for the sample images generated by AI (DALL-E 2 (https://openai.com/dall-e-2) and Stable Diffusion 1.5)^54^ for research purposes only.

### Step 2: Expressing emotions

Emotional self-expression, which refers to the verbal and non-verbal communication of one’s emotions, is another necessary skill in building emotional empowerment in children. Encouraging children to express their emotions in healthy ways is important for their emotional development and is a commonly used coping strategy that predicts better psychological and physical health in later life^55^. There are several ways that GAI could be used to help enhance a child’s ability to express their emotional state(s). For example, a GAI model could generate several images associated with specific emotions and provide corresponding labels or emotion words to help facilitate improved communication of a child’s emotional state (see Fig. 2). For some individuals, expressing their feelings through words can be challenging or insufficient. Creating personalized images may provide a new layer for engagement and a non-verbal medium through which expression of feelings can be communicated and practiced. This could also help facilitate a sense of relief or catharsis by permitting individuals to externalize and express their emotions and could provide several opportunities for individuals to identify feelings in themselves and others. As a variety of creative therapeutic approaches are used to help enable individuals to better express their emotions^56^, GAI may help enhance expressive therapeutic techniques through the development of more personalized and contextually relevant visual and auditory stimuli, facilitating unique and engaging avenues for emotional expression.

### Step 3: Managing emotions

Lastly, GAI may prove useful in improving a child’s ability to cope with various emotions through the provision of visual tools and resources to support emotional regulation. Broadly speaking, emotion regulation refers to the processes by which individuals can influence and exert control over their emotional state (i.e., which emotions they have, for how long as well as how strongly they experience and express them) and plays a vital role in young children’s emotional and cognitive development and later academic achievement^57,58^. The acquisition of emotion-regulation skills has been shown to improve the effectiveness of existing therapeutic interventions^58^. GAI may help foster improved emotional regulation through the creation of personalized coping resources. More specifically, idiosyncratic emotion-related content tailored to a child’s specific needs and experiences (e.g., coping cards, mood trackers, and emotion charts) could serve as visual reminders and tools to support one’s experience in learning to cope with various emotions. Incorporating additional evidence-based techniques (e.g., guided imagery, relaxation practices, and mindfulness), GAI could also be used to create individualized calming and soothing visuals, such as a peaceful natural scene or comforting images (e.g., lounging on a beach chair). These can be generated by collaboration between patient, provider and GAI, and eventually help bolster existing treatment strategies aimed at reducing distress and may also provide a powerful distraction in the face of aversive emotional states.

### Future of behavioral health support

**Fig. 3:**
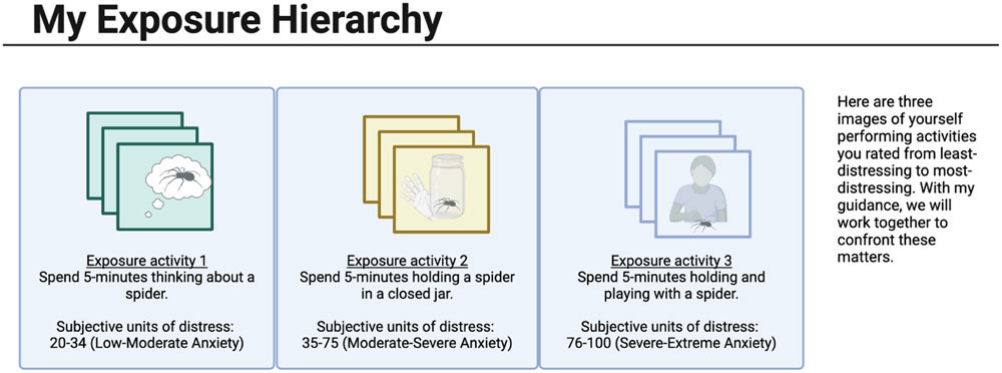
Emotion identification card with generated images. The card presents generated images of a patient reflecting anger, happiness, fear and sadness. The card shares instruction to guide patient to match images with emotions, and it guides to match images with corresponding emotional vocabulary items^79^.

Moving forward, the use of GAI-based applications may serve as a supportive tool in the delivery and/or implementation of a wide range of behavioral health treatments both during and outside of therapeutic settings. For example, GAI can enhance mindfulness-based interventions by creating visually and contextually relevant stimuli that facilitate relaxation, focus, and self-awareness. It can also be used to augment cognitive-behavioral therapies, such as exposure and response prevention through creating virtual environments or scenarios that mimic real-life situations that may trigger anxiety or phobias, allowing the patients to gradually expose themselves to specific fears in a controlled, convenient, and safe manner and learn coping skills to reduce associated distress (please see Fig. 3 and https://github.com/edsz7/supplementary for the sample images generated by AI for research purposes only). Broadly speaking, the personalization of various treatment modalities has been found to improve the effectiveness of the treatments, reduce heterogeneity in treatment outcomes, and better meet the needs of the patients^30^. Eventually, such personalization may help patients to be more compliant with out-of-session homework^59^. In addition, the automation of therapeutic materials across various interventions may reduce the need for manual content creation by providers, thus possibly saving time and potential costs associated with developing personalized materials.

Outside of more traditional psychotherapeutic approaches, creative therapy modalities, such as art and music therapy, may also benefit from the utilization of GAI for content generation (e.g., by producing tailored stimuli to evoke desired emotional and cognitive responses) and may be woven into existing creative therapeutic strategies (i.e., narrative strengths-based storytelling). As GAI models become more sophisticated and multimodal, they may be considered for facilitating and or supporting the development of novel therapeutic tools and approaches for behavioral health treatment, specifically for cases when it is hard to verbally communicate and express oneself.

GAI models could also assist in the provision of psychoeducation to help-seeking individuals by visually representing complicated concepts, processes, or emotions to improve understanding. Going beyond the clinical setting, the prospect of multimodal AI (e.g., image and video generation from voice inputs, text, or images) opens up new possibilities in the context of teletherapy and remote behavioral health services. Incorporated with virtual reality, extended reality, and augmented reality with LLMs, therapeutic services can also be expanded with conversational agents as personalized and guided self-therapy or self-training tools for patients^29^.

## Limitations, challenges, and considerations

Despite the benefits for healthcare service users and providers, this technology is not without potential limitations. In the context of the discussed use case, one limitation is the potential for generated images to lack authenticity and genuine emotional resonance. While GAI may be capable of producing visually and esthetically appealing images, it may struggle to capture or represent the necessary subtleties and complexities of human emotions accurately^60^, and it might lack the nuanced understanding and empathy that human therapists provide^61^. Therefore, unsupervised GAI applications (e.g., self-generated images, text, and audio) may currently pose a challenge and risk to be used in multimodal therapy delivery. These qualities are usually exhibited by a human therapist and cannot be entirely replicated by an AI. The non-judgmental listening and rapport building, that characterize successful therapy, may be impacted by the introduction of AI in terms of practice and assessment. Therefore, it is important to keep the provider in the loop for professional guidance and control of the process. We suggest that any GAI implementation should be thoughtfully designed to supplement rather than replace human interaction^35^, with studies needed to assess the impacts and outcomes on the therapeutic relationship. As a visual communication tool, GAI may require to be used via iterative or chain-of-thought process to visualize closer to what is being expressed as a part of AI-human collaboration^62,63^. A human feedback mechanism can also be used to train generative models to improve the outputs.

Building upon human-AI collaboration, inclusivity, and representativeness are necessary for equitable care delivery, and the AI-generated content should be relevant in terms of context and culture^64^. Emotional expression and interpretation can vary significantly across cultures, and what may be considered empowering in one culture might not hold the same meaning in another. Cultural inclusion could be a challenging task for providers (i.e., require training in inclusivity and culture) as well as GAI (e.g., require training datasets on a variety of populations, languages, and cultures). However, GAI could also be “prompted” to produce inclusive and individualized images based on user input. Thus, it is crucial that providers are trained and familiar with how to operate GAI with manual input and deploy GAI that is tuned to take into account cultural diversity and sensitivity to avoid reinforcing stereotypes or inadvertently causing harm (see https://github.com/edsz7/supplementary for a prompting example).

GAI and provider collaboration, rather than the use of GAI only in self-therapy, can also reduce encountering unexpected risks regarding patients safety and quality of care^35^. The AI-behavioral health convergence offers significant potential for care improvement. Therefore, we need to consider ethical implementations, particularly towards building privacy-preserving and trustworthy models, and supportive human interaction in therapeutic processes^65^. For instance, considering the potential bias and non-representative dataset (images, pictures, illustrations, figures, and annotations) used for GAI training, human-in-the-loop quality assurance, and content validation are necessary steps before using in practice^66^. Such practices may need to be defined or prescribed further by standards and guidelines towards GAI use and development, such as vetting of training datasets against biases as well as intellectual property right infringement^36,61^, and integrating fairness and equity approaches to ensure ethical and effective use in mental and behavioral health applications^67^. In line with that, an additional layer of transparency and accountability is needed in practice^36,68^, such as adding consent in clinical workflow protocol to disclose the risks and benefits of such approaches with patients, caregivers, and families, and adherence to medicolegal practices, data protection policies and legislations (e.g., HIPAA and GDPR)^61^. The number of government agencies and technology providers have been releasing guidelines^69^, regulations^70^, and call for action^71,72^ towards ethical and trustworthy GAI use and development, which could be informative toward GAI in behavioral healthcare practices.

The current use of AI as clinical tools or support systems so far has led to only a moderate improvement in cost-effectiveness^73^. Even though the marginal cost of AI-based decisions is minimal, its overall impact on net costs is unknown^74^. Therefore, the aforementioned considerations may further require investigation of operational effectiveness, including strategizing integration and workflow adjustment, system design updates, creating infrastructure (e.g., Cloud backend architecture and EHR integration) for deployment, planning of GAI-based practice reimbursement mechanisms, patient engagement testing and treatment adherence, developing patient and provider training curriculum to effectively use and incorporate GAI in practices, and cost-effectiveness analysis^75^.

In addition to the above considerations, the potential misuse of GAIs (e.g., deepfake) is a significant concern^76^. AI-generated images, if used unethically, can manipulate emotions, possibly causing psychological harm^77^. There could be instances where these powerful tools are used with malicious intent, such as promoting propaganda or spreading misinformation about behavioral health. It is critical to ensure that all data used by GAIs is appropriately anonymized and secured following standard encryption and cybersecurity practices. However, there could be a risk of identification of protected health information, especially with the use of patient audio and image data as input. A comprehensive framework of data governance should be in place to safeguard against any unauthorized access, misuse of data and handling data breach^61,78^. Regulatory frameworks and stringent monitoring processes need to be in place to prevent such misuse, ensuring that the power of GAIs is harnessed for beneficial purposes alone.

## Conclusions

In this paper, we highlight GAI impact on behavioral health, exemplifying image generation support on therapy towards recognizing, expressing, and managing emotions. The potential for integrating GAI models in therapeutic settings is open for exploration. GAI could be used for immersive therapeutic experiences leading to engaging therapy sessions, with personalized environments generated in real time based on the patient’s emotional state and therapeutic needs. Given the potential of GAI to transform behavioral healthcare, it is crucial for researchers and practitioners to collaborate in the development, evaluation, governance, and implementation of these tools and provide guidelines for practices and implications. By conducting rigorous, interdisciplinary research and trials at the intersection of behavioral health and GAI, we can ensure that these innovative solutions are optimally harnessed to improve patient care and promote overall well-being.
